# Genome dedoubling by DCJ and reversal

**DOI:** 10.1186/1471-2105-12-S9-S20

**Published:** 2011-10-05

**Authors:** Antoine Thomas, Jean-Stéphane Varré, Aïda Ouangraoua

**Affiliations:** 1 LIFL, UMR 8022 CNRS, Université Lille 1, INRIA Lille Nord Europe, Villeneuve d’Ascq, France

## Abstract

**Background:**

Segmental duplications in genomes have been studied for many years. Recently, several studies have highlighted a biological phenomenon called *breakpoint-duplication* that apparently associates a significant proportion of segmental duplications in Mammals, and the Drosophila species group, to breakpoints in rearrangement events.

**Results:**

In this paper, we introduce and study a combinatorial problem, inspired from the breakpoint-duplication phenomenon, called the *Genome Dedoubling Problem.* It consists of finding a minimum length rearrangement scenario required to transform a genome with duplicated segments into a non-duplicated genome such that duplications are caused by rearrangement breakpoints. We show that the problem, in the Double-Cut-and-Join (DCJ) and the reversal rearrangement models, can be reduced to an APX-complete problem, and we provide algorithms for the Genome Dedoubling Problem with 2-approximable parts. We apply the methods for the reconstruction of a non-duplicated ancestor of *Drosophila yakuba.*

**Conclusions:**

We present the *Genome Dedoubling Problem*, and describe two algorithms solving the problem in the DCJ model, and the reversal model. The usefulness of the problems and the methods are showed through an application to real Drosophila data.

## Introduction

Gene duplication is an important source of variations in genomes. Recently, several studies have highlighted biological evidence for abundant segmental duplications that occur around breakpoints of rearrangement events in the evolution of eukaryotes.

In mammals, an evidence for a strong association between duplications, genomic instability and large-scale chromosomal rearrangements in primate evolution was first reported in [1]. Later in [2], a study of all evolutionary rearrangement breakpoints between human and mouse genomes reported that 53% of the breakpoints were associated with segmental duplications, as compared to 18% expected in a random assignment of breaks. In [3], a first study of the human-mouse rearrangement breakpoints, considering only synteny blocks of length larger than 100Kb and duplicated sequences of length larger than 10Kb, showed that 25% (122/461) of the breakpoints contained duplicated sequences of length greater than 10kb.

The association between segmental duplications and regions of breaks of synteny was also reported in the Drosophila species group. In [4], an analysis of the breakpoints of *Drosophila yakuba* compared to two related species, *Drosophila simulans* and *Drosophila melanogaster*, revealed that the breakpoint regions of 59% of the reversals (17/29) were associated with inverted duplications of genes or other nonrepetitive sequences. Further evidences of the recurrent presence of repetitive sequences near breakpoints of rearrangement in the evolution of Drosophila were also reported in [5-7].

A rearrangement event is an operation that modifies the organization of a given genome by cutting the genome at some points called *breakpoints* to glue the exposed extremities in a different way. The biological phenomenon called *breakpoint-duplication* results in the presence of the same genomic segment on both extremities of a breakpoint in a rearrangement. Several biological models have been presented to explain the presence of duplicated sequences at rearrangement breakpoint regions. These models are based on DNA breaks repairs that produce duplicated segments because of staggered Single-Strand-Breaks [3,4], or non-reciprocal genetic exchange in Double-Strand-Breaks [5]. Most of these biological models support a nonrandom model of chromosomal evolution that implicates a predominance of recurrent small-scale duplications and large-scale evolutionary rearrangements within specific *fragile* regions. Moreover, the genetic instability of such regions is often suggested to be the cause rather than the consequence of duplicated genomic architecture [3,8]. Interestingly, a growing number of the breakpoint-duplications detected in Supra-primates evolution have also been linked to recurrent chromosomal rearrangements associated with common diseases in the human population [1-3,8,9]. In [10], breakpoint-duplications were also identified in humam sex chromosomes, allowing to order rearrangement events in time, based on the degree of divergence of the breakpoint-duplicated sequences.

In this paper, we are interested in using the segmental duplications of a given present-day genome that has undergone breakpoint-duplication rearrangements, in order to reconstruct a non-duplicated ancestral genome. We formally define the breakpoint-duplication phenomenon, and introduce a combinatorial problem called the *Genome Dedoublíng problem.* Given a genome that has undergone breakpoint-duplication rearrangements, possibly with other rearrangements events, the problem asks to find an ancestral genome such that the number of rearrangement events needed to tranform the ancestor into the given genome is minimal. Note that the Genome Dedoubling problem asks to find a non-dupliated ancestor of a given duplicated genomes, as the *Genome Halving problem* introduced in [11] that consists of, given a genome that has undergone a whole-genome duplication followed by rearrangement events, finding the ancestral genome that was present right before the whole-genome duplication event. However, the two problems and their solutions are different as they aim at recovering different types of duplication events, breakpoint-duplications and whole-genome duplications. As the Genome Halving problem is motivated by the whole-genome duplication events in molecular evolution, the Genome Dedoubling problem is motivated by breakpoint-duplication events in molecular evolution. Both problems are useful for the comparison of genomes with duplicated segments.

In the following, we study the Genome Dedoubling problem under the Double-Cut-and-Join (DCJ) and the reversal rearrangement models. In Section **Methods**, we formally present breakpoint-duplication (BD) rearrangements and the Genome Dedoubling Problem. We show that the problem can always be regarded as a Dedoubling Problem on totally duplicated genomes. In Section **Genome dedoubling by DCJ**, we study the problem under the DCJ model, on multichromosomal then unichromosomal genomes. We prove the NP-completeness of the problems by reduction to an APX-complete problem, and provide algorithms with a linear time complexity, except for an APX-complete part that is 2-approximable. In Section **Genome dedoubling by reversal**, we study the problem under the reversal model on oriented genomes, making use of some results of the Hannenhalli-Pevzner (HP) theory [12] on sorting by reversal described in [13,14]. We provide an algorithm with a quadratic time complexity, except for an APX-complete part that is 2-approximabe. In Section **Application,** an application for the reconstruction of a non-duplicated ancestor of *Drosophila yakuba*, using data from [4], is presented.

## Methods

In this section we give the main definitions and notations of duplicated genomes and rearrangements. Next, we generalize the definitions of rearrangements in order to introduce a formal definition of *breakpoint-duplication rearrangements*, and the *Genome Dedoubling Problem* studied in the paper.

### Duplicated genomes

A genome consists of linear or circular chromosomes that are composed of genomic markers. Markers are represented by signed integers such that the sign indicates the orientations of markers in chromosomes. By convention, – –*x* = *x.* A linear chromosome is represented by an ordered sequence of signed integers surrounded by the unsigned marker ○ at each end indicating the telomeres. A circular chromosome is represented by a circularly ordered sequence of signed integers. For example, (1 2 –3) (○ 4 –5 ○) is a genome composed of one circular and one linear chromosome.

Each genome contains at most two occurrences of each marker. Two copies of a same marker in a genome are called paralogs. If a marker *x* is present twice, one of the paralogs is represented by . By convention, . Here, such markers represent segments duplicated by a breakpoint-duplication rearrangement.

**Definition 1 ***A* duplicated genome *is a genome in which a subset of the markers are duplicated.*

For example,  is a duplicated genome where markers 1, 2, and 5 are duplicated. A *non-duplicated genome* is a genome in which no marker is duplicated. A *totally duplicated genome* is a duplicated genome in which all markers are duplicated. For example,  is a totally duplicated genome.

An *adjacency* in a genome is a pair of consecutive markers. Since a genome can be read in two directions, the adjacencies (*x y*) and (*–y –x*) are equivalent. For example, the genome  has seven adjacencies, , and .

**Definition 2*** A* dedoubled genome *is a duplicated genome G such that for any duplicated marker x in G*, *either*, *or**is an adjacency of G.*

For example,  is a dedoubled genome. The *reduction* of a dedoubled genome *G*, denoted by *G^R^*, is the genome obtained from *G* by replacing every pair , or  by a single marker *x.* For example the reduction of  is *G^R^* = (1 –2) (○ –3 4 –5 ○).

### Rearrangement

A rearrangement operation on a given genome cuts a set of adjacencies of the genome called *breakpoints* and forms new adjacencies with the exposed extremities, while altering no other adjacency. In this paper, we consider two types of rearrangement operation called *double-cut-and-join* (*DCJ*) and *reversal.* In the sequel, the breakpoints of a rearrangement operation are indicated in the genome by the symbol _▲_, and the new adjacencies are indicated in the genome by dots.

A *DCJ* operation on a genome *G* cuts two different adjacencies in *G* and glues pairs of the four exposed extremities to form two new adjacencies. For example, the following DCJ cuts adjacencies (1 2) and  to produce  and (–5 2).

A *reversal* on a genome *G* is a DCJ operation that cuts two adjacencies (*a b*) and (*c d*) in a chromosome of *G* of the form (… *a b* … *c d* …) to form two new adjacencies adjacencies (*a* –*c*) and (–*b d*), thus reversing the orientation of the segment of *G* beginning with marker *b* and ending with marker *c*. For example, the following reversal cuts adjacencies  and  and reverses the segment .

A *DCJ* (*resp. reversal*) *scenario* between two genomes *A* and *B* is a sequence of DCJ (resp. reversal) operations allowing to transform *A* into *B.* The length of a scenario is the number of rearrangement operations composing the scenario.

The *DCJ* (*resp. reversal*) *distance* between two genomes *A* and *B* is the minimum length of a DCJ (resp. reversal) scenario between *A* and *B.*

### Breakpoint-duplication rearrangements

We now generalize the definitions of rearrangement operations to account for possible duplications at their breakpoints.

A *1-breakpoint-duplication DCJ* (1-BD-DCJ) operation on a genome *G* is a rearrangement operation that alters two different adjacencies (*a b*) and (*c d*) of *G*, by:

• first adding markerat the appropriate position to produce segment ,

• then applying a DCJ operation that cuts adjacencies  and (*c d*) to produce either (*a d*) and , or (*a* –*c*) and .

A *2-breakpoint-duplication DCJ* (2-BD-DCJ) operation on a genome *G* is a rearrangement operation that alters two different adjacencies (*a b*) and (*c d*) of *G*, by:

• first adding markers  and  at the appropriate positions to produce segments  and ,

• then applying a DCJ operation that cuts adjacencies  and  to produce either  and , or (*a* –*c*) and .

**Definition 3 ***A* breakpoint-duplication DCJ (*BD-DCJ*) *operation on a genome G is either a 1-BD-DCJ operation*, *or a 2-BD-DCJ operation.*

In the sequel, if some markers are duplicated by a BD-DCJ operation, they are indicated in bold font in the initial genome. For example, the following rearrangement is a 2-BD-DCJ operation that acts on adjacencies (–2 –1) and (4 –3), and duplicates markers 2 and 4. The intermediate step resulting in the duplication of markers 2 and 4 is shown above the arrow.

To summarize, a BD-DCJ operation consists of a *first step* in which one or two markers are duplicated, followed by a *second step* where a DCJ operation is applied. Similarly, we now define a *breakpoint-duplication reversal* (BD-reversal) operation.

**Definition 4 ***A* breakpoint-duplication reversal (*BD-reversal*) *operation on a genome G is a BD-DCJ operation such that the DCJ operation applied in the second step of the BD-DCJ operation is a reversal.*

For example, the following rearrangement is a BD-reversal that is a 1-BD-DCJ operation that acts on adjacencies (2 –1) and (–3 4), and duplicates marker 2.

A *BD-DCJ scenario* (resp. *BD-reversal scenario*) between a non-duplicated genome *A* and a duplicated genome *B* is a sequence composed of BD-DCJ (resp. BD-reversal) operations and possibly DCJ (resp. reversal) operations allowing to transform *A* into *B.*

**Definition 5 ***Given a non-duplicated genome A and a duplicated genome B*, *the* BD-DCJ distance (*resp.* BD-reversal distance) *between A and B is the minimal length of a BD-DCJ* (*resp. BD-reversal*) *scenario between A and B.*

We now give an obvious, but useful property allowing to reduce a BD-DCJ scenario to a DCJ scenario.

**Proposition 1*** Given a non-duplicated genome A and a duplicated genome B*, *for any a BD-DCJ* (*resp. BD-reversal*) *scenario between A and B*, *there exists a DCJ* (*resp. reversal*) *scenario of same length between a dedoubled genome D and B such that the reduction of D is A* (*D^R^* = *A*)*.*

**Proof.** Let *S* be a BD-DCJ (resp. BD-reversal) scenario between *A* and *B. D* is the genome obtained from *A*, by adding, for any marker *x* duplicated by a BD-DCJ operation in *S*, the marker  in a way to produce either adjacency , or  as done in *S.* Thus, *D^R^* = *A.* The DCJ (resp. reversal) scenario between *D^R^* and *B* having the same length as *S*, is the sequence of DCJ (resp. reversal) contained in *S* or in BD-DCJ (resp. BD-reversal) operations of *S*, with the same order as in *S.* ■

For example, in the following, a BD-reversal scenario of length 4 between *A* = (○ 1 2 3 4 5 ○) and  induces a reversal scenario of length 4 between  and *B.*

### Genome dedoubling problem

We now state the genome dedoubling problems considered in this paper.

**Genome dedoubling problem:*** Given a duplicated genome G*, *the* DCJ (resp. reversal) genome dedoubling problem *consists of finding a non-duplicated genome H such that the BD-DCJ* (*resp. BD-reversal*) *distance between H and G is minimal.*

Given a duplicated genome *G*, we denote by *d_dcj_*(*G*) (resp. *d_rev_*(*G*)), the minimum BD-DCJ (resp. BD-reversal) distance between any non-duplicated genome and *G.* From Proposition 1, the following proposition is straightforward.

**Proposition 2 ***Given a duplicated genome G*, *the DCJ* (*resp. reversal*) *genome dedoubling problem on G is equivalent to finding a dedoubled genome D such that the DCJ* (*resp. reversal*) *distance between D and G is minimal.*

The next proposition describes a further reduction of the genome dedoubling problem on a duplicated genome *G.*

**Proposition 3 ***Given a duplicated genome G*, *the* DCJ (resp. reversal) genome dedoubling problem *on G is equivalent to the* DCJ (resp. reversal) genome dedoubling problem *on the totally duplicated genome G^T^ obtained from G by replacing every maximal subsequence of non-duplicated markers beginning with a marker x by the pair*.

**Proof.** See proof in Additional file 1 (Supplemental proofs). ■

For example, solving the DCJ (resp. reversal) genome dedoubling problem on  is equivalent to solving it on . The transformations applied on *G* to obtain *G^T^* are indicated in bold font.

In the sequel, *G* will always denote a totally duplicated genome, and we focus in Sections **Genome dedoubling by DCJ** and **Genome dedoubling by reversal** on the problem of finding a dedoubled genome *D* such that the DCJ (resp. reversal) distance between *D* and *G* is minimal.

## Results

In this section, we first study the Genome Dedoubling Problem under the DCJ model. Next, we study the problem under the reversal model on oriented genomes described in the Hannenhalli-Pevzner (HP) theory on sorting by reversal [12-14].

### Genome dedoubling by DCJ

In this section, *G* denotes a totally duplicated genome. In order to give a formula for the DCJ dedoubling distance of *G*, *d_dcj_*(*G*), we use a graph called the *dedoubled adjacency graph* of *G.*

#### Dedoubled adjacency graph

**Definition 6*** The* dedoubled adjacency graph *of G*, *denoted by*, *is the graph whose vertices are the adjacencies of G*, *and for any marker x there is one edge between the vertices* (*x u*) *and*, *and one edge between the vertices* (*y x*) *and**.*

**Figure 1:**
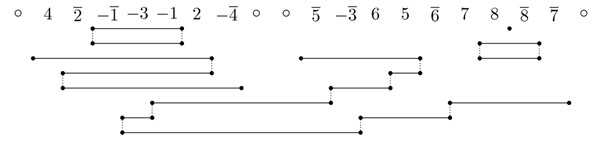
The adjacency graph of .

An example of dedoubled adjacency graph is depicted in Fig. 1. In the following, we will simply refer to dedoubled adjacency graphs as adjacency graphs.

Note that all vertices in  have degree one or two. Thus, the connected components of  are only paths and cycles. These paths and cycles are called *elements* of .

Given a couple of paralogous markers , an element of the graph  is said to *contain* the couple  if it contains the edge linking vertices (*x u*) and , or the edge linking vertices (*y x*) and . By definition, a couple  can possibly be contained in only one element *A* of  if element *A* contains both edges  and . In this case, *A* is said to *contain twice* the couple , and *A* is called a *duplicated* element of . If an element *A* contains no couple  twice, then it is called a *non-duplicated* element of . If the two edges  and  belong to two different elements *A* and *B* of , then *A* and *B* will both contain . In this case, we say that *A* and *B intersect.* If two elements *A* and *B* do not intersect, then we say that *A* and *B* are *independent.* For example in Fig. 1 the two paths of the adjacency graph are duplicated, while the three cycles are non-duplicated. The leftmost path and the leftmost cycle intersect because they both contain the couple , while the two paths are independent. Given an element *A* of , the *set induced by A* is the set of couples  contained in *A.*

#### General sorting

In this section, we prove the following theorem:

**Theorem 1*** Let n be the number of couples of paralogous markers in G. Let C_i_ be the maximum size of a subset of non-duplicated pairwise independent cycles in**. The DCJ dedoubling distance of G is d_dcj_*(*G*) = *n – C_i_.*

For example, in Fig. 1, the maximum size of a subset of non-duplicated pairwise independent cycles is 2 as there are three cycles, and the two rightmost cycles intersect. The distance would then be *d_dcj_*(*G*) = 8 – 2 = 6. To prove Theorem 1, we use the following properties:

**Property 1 ***Let n be the number of couples of paralogous markers in G.*

*1. The maximum size C_i_ of a set of non-duplicated pairwise independent cycles in the graph**is n.*

*2. If G is dedoubled genome*, *then**contains n non-duplicated pairwise independent cycles*, *each containing a single couple of paralogous markers*, *plus possibly other cycles. In this case*, *C_i_* = *n.*

*3. A DCJ operation can only alter the maximum size C_i_ of a set of non-duplicated pairwise independent cycles by* –1, 0 *or* +1.

**Proof.** See proof in Additional file 1 (Supplemental proofs). ■

Algorithm 1 is an algorithm that provides a *n* – *C_i_* length DCJ scenario transforming *G* into a dedoubled genome.

We now have all the pre-requisites to give the proof of Theorem 1. The proof can be found in Additional file 1 (Supplemental proofs).

**Lemma 1*** Choosing a maximum size set of non-duplicated pairwise independent cycles in**is an APX-complete problem*, *approximable with an approximation ratio of 2.*

**Proof.** See proof in Additional file 1 (Supplemental proofs). ■

From Lemma 1, the complexity of the Genome Dedoubling problem by DCJ follows immediately.

**Corollary 1*** The Genome Dedoubling problem by DCJ is NP-complete. Algorithm 1 solves the problem in linear time complexity*, *except for the computation of the set of cycles S_i_ that is 2-approximable.*

#### Sorting between linear unichromosomal genomes

In this section, we search for a minimum length DCJ scenario that transforms a duplicated genome consisting of a single linear chromosome into a dedoubled genome consisting of a single linear chromosome. The results of this section will then be used in the next section for the study of the Genome Dedoubling problem under the reversal model.

In this section and the sequel, *G* denotes a totally duplicated genome consisting of a single linear chromosome. In this case, the graph  contains exactly one path, and possibly several cycles.

**Definition 7*** The path in**is said to be* valid *if it contains every couple**of paralogous markers in G.*

A DCJ operation that merges a cycle *c* of in the path *p* is a DCJ operation that acts on an adjacency of *c* and an adjacency of *p*, thus gathering c and *p* into a longer path.

Note that if *G* is a dedoubled genome, then the path in  is necessarily valid. We call such a genome a *dedoubled linear genome.* So, if the path in  is not valid, then any DCJ scenario transforming *G* into a dedoubled linear genome will merge, in the path, cycles containing the couples  that are not contained in the path.

In the following, we always denote by *m* the minimum number of cycles required to make the path of  valid. We also denote by *C_i_* the maximum size of a subset of non-duplicated pairwise independent cycles. First, we have the following property:

**Property 2*** Let C be the number of cycles in**. We have C_i_* = *C – m.*

**Proof.** See proof in Additional file 1 (Supplemental proofs). ■

From Property 2, we then have the following lemma.

**Lemma 2*** Let n be the number of couples of paralogous markers in G. Let C be the number of cycles in**. The minimum length d of a DCJ scenario transforming G into a dedoubled genome consisting of a single linear chromosome equals d* = *n – C* + 2*m.*

**Proof.** See proof in Additional file 1 (Supplemental proofs). ■

From Property 2 and Lemma 2, we immediately have the following complexity.

**Corollary 2*** The problem of finding a DCJ scenario transforming G into a dedoubled genome consisting of a single linear chromosome is NP-complete. Algorithm 1*, *in which we add the line* (*2’: Merge in the path all the cycles that are not in S_i_*) *between lines* 2 *and* 3, *solves the problem in linear time complexity*, *except for the computation of the set of cycles S_i_ that is 2-approximable.*

### Genome dedoubling by reversal

We build and use a graph that behaves like the *arc overlap graph* used in [13] for the Hannenhalli-Pevzner theory of sorting by reversal [12]. The genome *G* consists of a single linear chromosome.

#### Dedoubled overlap graph

For any couple  of paralogous markers in *G*, the segment of  is the smallest segment of *G* containing both markers *x* and . For example, in genome , the segment of  is , and the segment of  is .

**Definition 8*** The* dedoubled overlap graph *of G*, *denoted by*, *is the graph whose vertices are all the couples**of paralogous markers of G*, *and there is an edge between two vertices**and**if the segments of u and v overlap.*

An example of dedoubled overlap graph is depicted in Fig. 4.a. In the following, we will simply refer to dedoubled overlap graphs as overlap graphs.

**Figure 2:**
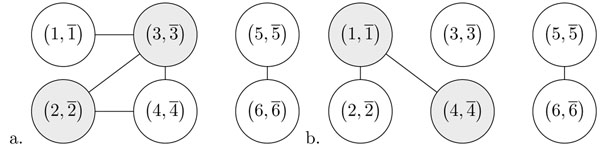
a. The overlap graph of . Oriented vertices are colored in grey. The graph  has two connected components, one oriented and one unoriented. b. the overlap graph obtained after applying the reversal  to produce adjacency.

**Figure 3:**
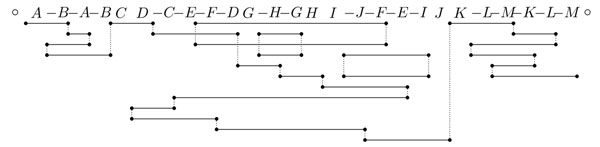
The adjacency graph of *G* = (○ *A –B –A –B C D –C –E –F –D G –H –G H I –J –F –E –I J K –L –M –K –L –M* ○).

**Figure 4:**
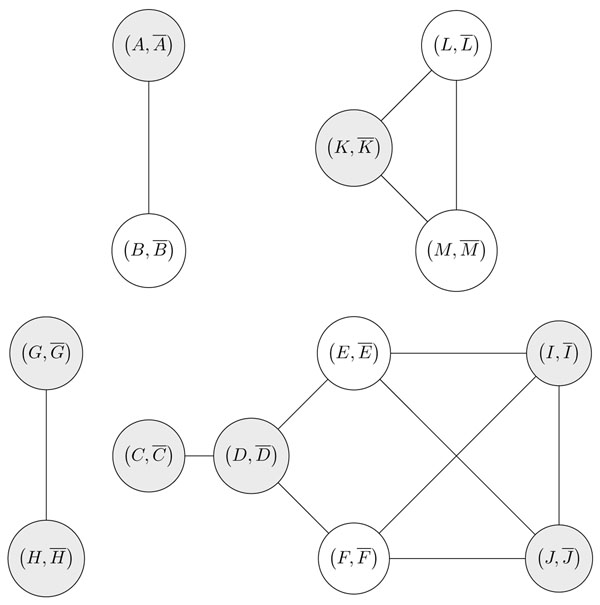
The overlap graph of *G* = (○ *A –B –A –B C D –C –E –F –D G –H –G H I –J –F –E –I J K –L –M –K –L –M* ○). Oriented vertices are colored in grey. The graph has 4 oriented connected components.

The vertex  of the graph  is *oriented* if *x* and  have different signs in *G*, otherwise it is *unoriented.* If the vertex  is oriented then there exists a reversal operation denoted by  that produces the adjacency  and a reversal operation denoted by  that produces the adjacency *.* For example, in genome  is an oriented vertex of .

The overlap graph of *G* behaves like arc overlap graphs used in [13] for the Hannenhali-Pevzner theory of sorting by reversal [12]. Indeed, given an oriented vertex  of the graph , performing the reversal  or  complements the subgraph induced by  and all its neighbouring vertices, and changes the orientation of all vertices in this subgraph (see Fig. 4.b).

A connected component of the graph  is *oriented* if it contains at least one oriented vertex, otherwise it is *unoriented.* A genome is *oriented* if all connected components of the graph  are oriented, otherwise it is *unoriented.*

Given an oriented vertex  of the graph , the score of  is the number of oriented vertices in the overlap graph of the genome obtained after applying  on *G.* Note that the same number of oriented vertices is obtained after applying  on *G.*

**Property 3*** Let**be an oriented vertex of**of maximum score. Performing**or**does not create new unoriented connected components in the overlap graph of the genome obtained.*

**Proof.** See proof in Additional file 1 (Supplemental proofs). ■

In the sequel, we focus on sorting oriented genomes using reversal dedoubling scenarios. A totally duplicated genome *G* consisting of a single linear chromosome is called a *valid-path genome* if the single path in  is valid. Otherwise, it is called a *non-valid-path genome.*

#### Sorting an oriented valid-path genome

In this section, we consider an oriented valid-path genome *G.* With *n* being the number of couples of paralogous markers in *G*, we have the following theorem:

**Theorem 2*** Let G be an oriented valid-path genome. Let C be the number of cycles in**. The reversal dedouhling distance of G is d_rev_*(*G*) = *n – C.*

**Proof.** See proof in Additional file 1 (Supplemental proofs). **■**

#### Sorting an oriented non-valid-path genome

In this section, *G* denotes an oriented non-valid path genome. At least *m* cycles of  have to be merged in the path to make it valid.

An edge  or  of the adjacency graph  is called oriented if markers *x* and  have different signs. Note that extracting a cycle from any element of the graph  requires this element to contain oriented edges. It is easy to see that given two adjacencies picked in a given element, a reversal acting on these adjacencies will extract a cycle if and only if the path linking these adjacencies contains an odd number of oriented edges. Thus, we have the following lemma:

**Lemma 3 ***Let G be an oriented non-valid-path genome. Merging a cycle of**in its path never creates unoriented connected components in the overlap graph of the genome obtained.*

**Proof.** See proof in Additional file 1 (Supplemental proofs). ■

**Theorem 3 ***Let G be an oriented non-valid-path genome. Let C be the number of cycles in the graph**and m be the minimum number of cycles to merge in the path to make it valid. The reversal dedoubling distance of G is d_rev_*(*G*) = *n – C* + 2*m*.

**Proof.** See proof in Additional file 1 (Supplemental proofs). ■

Prom Lemma 1 and Property 2, the complexity of the Genome Dedoubling problem by reversal on oriented genomes follows immediately.

**Corollary 3*** The Genome Dedoubling problem by reversal on oriented genomes is NP-complete. Algorithm 2 solves the problem in quadratic time complexity*, *except for the computation of S_i_ that is 2-approximable.*

## Application

We applied Algorithm 2 to reconstruct an ancestral chromosome for the chromosome 2 of *Drosophila yakuba* using a dataset obtained from [4] with *Drosophila melanogaster* used as the outgroup. The results obtained are in good agreement with the biological results explaining the evolution of the chromosome 2 from *Drosophila yakuba* to *Drosophila melanogaster* in the litterature [4,15]. See Additional file 2 (Experimental results) for a description of the dataset and the results of the application.

## Conclusion

In this paper, we introduced the genome dedoubling problem in the DCJ rearrangement model, NP-complete in both the multichromosomal and the linear unichromosomal case, by reduction to an APX-complete problem. For both cases, we described an algorithm solving the problems in linear time complexity, except for an APX-complete part that is 2-approximable. We also presented some results on the Genome Dedoubling problem by reversal, providing an algorithm solving the problem on oriented genomes in quadratic time complexity, except for an APX-complete part that is 2-approximable. The case of unoriented genomes in the reversal model will be treated in a future paper. Unsurprisingly, partial results obtained so far tend to show that the general distance formula can be written as *d_rev_*(*G*) = *n – C* + 2*m* + *t*, with *t* corresponding to the cost of genome orientation. However, the cost *t* here differs from the orientation cost described in the classical reversal theory based on the unoriented component tree [14]. In our case, the structure of the graph  allows to orient components while not decreasing the number of cycles, or even increasing it in some cases. This requires proper identification of different kinds of merging reversals and further extensions on the data structures presented in this paper.

The second obvious extension of the present work, as in the the Genome Halving problem theory [16], is to generalize the Genome Dedoubling problem defined on a single genome, to the *Guided Genome Dedoubüng problem*, that asks to find a non-duplicated genome that minimizes the breakpoint-duplication distance to a given duplicated genome, plus the distance to a given non-duplicated genome. A further extension of this work consists of taking account of the degree of divergence of the breakpoint-duplicated sequences to order the rearrangement operations in time as done in [10].

## Competing interests

The authors declare that they have no competing interests.

## Authors' contributions

The work was divided in four steps: 1) Formal introduction and reduction of the Genome Dedoubling problem. 2) Design of the study. 3) Study of the Genome Dedoubling problem by DCJ. 4) Study of the Genome Dedoubling problem by reversal on oriented genomes. AT participated in 2) and 3), and carried out 4). JSV participated in 2), 3) and 4). AO carried out 1), 2) and 3), and participated in 4). All authors participated in writing the manuscript, and approved the final manuscript.

## Supplementary Material

Additional file 1 **Supplemental proofs** Additional file 1 is a PDF file containing the proofs of Proposition 3, Property 1, Lemma 1, and Property 3. Click here for file

Additional file 2 **Experimental results** Additional file 2 is a PDF file containing a description of an application of the methods to real Drosophila data. Click here for file
