# Supplementary material for: Genome dedoubling by DCJ and reversal
Source: BMC Bioinformatics. 2011 Oct 5;12(Suppl 9):S20. doi: 10.1186/1471-2105-12-S9-S20 (PMC3283308; doi:10.1186/1471-2105-12-S9-S20)
Supplement: Additional file 1 — Supplemental proofs Additional file 1 is a PDF file containing the proofs of Proposition 3, Property 1, Lemma 1, and Property 3. [file 1471-2105-12-S9-S20-S1.pdf]

Additional Files

Additional file 1 — Supplemental proofs

Additional file 1 is a PDF file containing the proofs of Proposition 3, Property 1, Lemma 1, and Property 3.

Proof of Proposition 3

Let D be a dedoubled genome. We show that D can be transformed into a dedoubled genome D' containing all adjacencies of G^T of the type $(x\ \ \overline{x})$, such that the DCJ (resp. reversal) distance between G^T and D' is less or equal to the DCJ (resp. reversal) distance between G^T and D.
For any adjacency $(x\ \overline{x})$ of G^T that is not contained in D, D necessarily contains the adjacency $(\overline{x}\ \ x)$, since D is a dedoubled genome. The genome D' is obtained by successively replacing the segment $(\overline{x}\ \ x)$ in D by the segment $(x\ \ \overline{x})$, for any adjacency $(x\ \overline{x})$ in G^T that is not contained in D. By construction of D' from D, it follows immediately that the DCJ (resp. reversal) distance between G^T and D' is less or equal to the DCJ (resp. reversal) distance between G^T and D. Thus, there exists a dedoubled genome containing all adjacencies of G^T of the type $(x\ \overline{x})$ that minimizes the DCJ (resp. reversal) distance to G^T. Therefore, there exists an optimal scenario that preserves the consecutivity of unduplicated markers grouped into a subsequence.

Proof of Property 1

The first property comes from the fact that each couple $(x, \overline{x})$ is contained in at most one cycle of a set of pairwise independent cycles. The second property is due to the fact that, by definition, a dedoubled genome always contains either the adjacency $(x\ \overline{x})$ or the adjacency $(\overline{x}\ x)$ for any couple $(x, \overline{x})$. The last property is due the fact that a DCJ operation acts on two adjacencies, cutting them in order to form two new adjacencies. Then, a DCJ either extract a new cycle that contributes to increase C_i by 1, or destroy a single cycle in any set of non-duplicated pairwise independent cycles, thus decreasing C_i by 1, or leaves C_i unchanged.

Proof of Theorem 1

From Property 1, a DCJ operation cannot increase C_i by more than 1. Algorithm 1 provides a DCJ scenario transforming G into a dedoubled genome, by increasing C_i by 1 at each DCJ operation until it reaches its upper bound n. Algorithm 1 then provides a minimum length scenario which is of length $n - C_i$.

Proof of Lemma 1

We show the equivalence of the Maximum non-duplicated pairwise independent cycles problem with a *2-frequency Maximum Packing Set problem* known to be APX-complete [17] and 2-approximable [18]. A *2-frequency collection of sets* is a collection of finite sets such that each element of any set belongs to at most two sets of the collection. Given a 2-frequency collection C_n of sets, the 2-frequency Maximum Packing Set problem on C_n asks to find the maximum number of pairwise disjoint sets in C_n.

Let C_n be the collection of sets of couples induced by non-duplicated cycles of $\mathcal{A}(G)$. Computing the maximum size C_i of a subset of non-duplicated pairwise independent cycles in $\mathcal{A}(G)$ can obviously be reduced to the *2-frequency Maximum Packing Set problem* on C_n. Conversely, given a 2-frequency collection C_n of sets, containing elements of the form $(x, \overline{x})$ without loss of generality, Algorithm 3 builds a totally duplicated genome G such that the non-duplicated cycles of $\mathcal{A}(G)$ induce the sets of C_n.

Algorithm 3 produces a genome G such that all cycles of the adjacency graph $\mathcal{A}(G)$ are non-duplicated, and each of these cycles induces a set of the 2-frequency collection C_n.

Proof of Property 2

Let $\mathcal{S}$ be the set of all cycles from G ($|\mathcal{S}| = C$). If $\mathcal{S}_m$ is a set of cycles that can be merged in the path to make it valid, then $\mathcal{S} - \mathcal{S}_m$ is a set of non-duplicated pairwise independent cycles because of the two facts that follows. 1. Any cycle c contained in $\mathcal{S} - \mathcal{S}_m$ is non-duplicated, otherwise the path obtained after the mergings would not be valid as it would not contain any couple of paralogous markers $(x, \overline{x})$ contained twice in c. 2. Any two cycles c_1 and c_2 of $\mathcal{S} - \mathcal{S}_m$ are independent, otherwise the path obtained after the mergings would not be valid as it would not contain any couple of paralogous markers $(x, \overline{x})$ contained in both c_1 and c_2.

Proof of Lemma 2

First, from Property 2, we have $n - C + 2m = n - C_i + m$. Similarly to C_i, a DCJ operation can only alter m by $+1$, -1 or 0. Next, a DCJ operation that increases C_i by 1 also increases C by 1, and then leaves m unchanged. Conversely, a DCJ operation that decreases m by 1 leaves C_i unchanged.

Algorithm 1 in which we add the line (2': Merge in the path all the cycles that are not in S_i) between lines 2 and 3 provides a DCJ scenario that first decreases m until it reaches its lower bound 0 (in m DCJ operations), then increases C_i until it reaches its upper bound n (in $n - C_i$ DCJ operations).

Algorithm 3 Reducing an instance of the 2-frequency Maximum Packing Set problem into an instance of the Maximum non-duplicated pairwise independent cycles problem.

```
G = empty genome
for any element (x, \overline{x}) in the sets of C_n do
    Visited(x, \overline{x}) = False
    Add in G two chromosomes (∘ x ∘) and (∘ \overline{x} ∘)
end for
for any set S = {(x_1, \overline{x_1}), (x_2, \overline{x_2}), ..., (x_k, \overline{x_k})} in C_n do
    if Visited(x_1, \overline{x_1}) == False then
        (a, b) = (x_1, \overline{x_1});
        Visited(x_1, \overline{x_1}) = True
    else
        (a, b) = (\overline{x_1}, x_1)
    end if
    if k == 1 then
        Glue adjacencies (a ∘) and (∘ b) to form adjacency (a b)
    else
        start = a
        for i : 2 → k − 1 do
            if Visited(x_i, \overline{x_i}) == False then
                (u, v) = (x_i, \overline{x_i})
                Visited(x_i, \overline{x_i}) = True
            else
                (u, v) = (\overline{x_i}, x_i)
            end if
            Glue adjacencies (u ∘) and (∘ b) to form adjacency (u b)
            (a, b) = (u, v)
        end for
        if Visited (x_k, \overline{x_k}) == False then
            (u, v) = (x_k, \overline{x_k})
            Visited(x_k, \overline{x_k}) = True
        else
            (u, v) = (\overline{x_k}, x_k)
        end if
        Glue adjacencies (u ∘) and (∘ b) to form adjacency (u b)
        Glue adjacencies ((start ∘) and (∘ v) to form adjacency (start v)
    end if
end for
```

Proof of Property 3

The proof is the same as the proof of Theorem 10 in [13]: if Rev($x \;\; \overline{x}$) or Rev($\overline{x} \;\; x$) creates a new unoriented connected component C in the overlap graph. Then, C necessarily contains a vertex $(w, \overline{w})$ that was adjacent to $(x, \overline{x})$ and oriented before applying Rev($x \;\; \overline{x}$) or Rev($\overline{x} \;\; x$), and such that the score of $(w, \overline{w})$ was greater than the score of $(x, \overline{x})$, which is a contradiction.

Proof of Theorem 2

In Algorithm 2, since G is a valid path genome, then S_i is the set of all cycles. Thus, Algorithm 2 provides a reversal scenario of length $n - C$, which is the smallest length that can be reached since $d_{dcj}(G) = n - C_i = n - C + m = n - C$ as $m = 0$ here, and we always have $d_{dcj}(G) \leq d_{rev}(G)$ as a reversal scenario is a DCJ scenario.

Proof of Lemma 3

A *component* of G is the smallest segment of G containing all markers of a connected component in $\mathcal{O}(G)$. Breakpoints of merging reversals can always be inside two distinct components of the genome. Performing the operation gathers these two components of the genome, possibly with some other as well, into a single component, obviously containing all couples of paralogous markers that used to be in the merged components. Therefore, it has to be an oriented component. Assuming otherwise, it would be possible to perform the inverse operation in the resulting genome, extracting a cycle from an unoriented component.

Proof of Theorem 3

From Lemma 2, we have that $d_{rev}(G) \geq n - C + 2m$ as $d_{rev}(G) \geq d_{dcj}(G)$. Algorithm 2 provides a scenario of length $n - C_i + m = n - C + 2m$.
