# Supplementary material for: Genome dedoubling by DCJ and reversal
Source: BMC Bioinformatics. 2011 Oct 5;12(Suppl 9):S20. doi: 10.1186/1471-2105-12-S9-S20 (PMC3283308; doi:10.1186/1471-2105-12-S9-S20)
Supplement: Additional file 2 — Experimental results Additional file 2 is a PDF file containing a description of an application of the methods to real Drosophila data. [file 1471-2105-12-S9-S20-S2.pdf]

Additional file 2 — Experimental results

Additional file 2 is a PDF file containing a description of an application of the methods to real Drosophila data.

We applied Algorithm 2 to reconstruct an ancestral chromosome for the chromosome 2 of *Drosophila yakuba* (*D.yak*) (Muller's Elements B and C). The dataset is based on 14 synteny blocks shared with the chromosome 2 of *Drosophila melanogaster* (*D.mel*), and 13 duplicated segments identified at breakpoints in (*D.yak*) [4]. Figure 3 and Table S5 in [4] provide the following arrangement of the synteny blocks, numbered from 14 to 36 according to their order in *D.mel*, and the 13 duplicated segments (numbered from A to M) in the chromosome 2 of *D.yak*:

$(\circ\ 14\ A\ {-17}\ 19\ {-B}\ {-15}\ {-A}\ 18\ {-16}\ {-B}\ 20\ C\ 27\ D\ {-21}\ {-C}\ {-26}\ {-E}\ {-F}\ {-28}\ {-D}\ 22\ G\ {-H}\ {-23}\ {-G}\ H$
$24\ I\ {-J}\ {-29}\ {-F}\ {-E}\ {-25}\ {-I}\ J\ 30\ K\ {-33}\ {-L}\ 35\ {-M}\ {-31}\ {-K}\ 34\ {-L}\ {-32}\ {-M}\ 36\ \circ)$.

After discarding the synteny blocks from the sequence, the following totally duplicated sequence with $n = 13$ duplicated markers is obtained:

$G = (\circ\ A\ {-B}\ {-A}\ {-B}\ C\ D\ {-C}\ {-E}\ {-F}\ {-D}\ G\ {-H}\ {-G}\ H\ I\ {-J}\ {-F}\ {-E}\ {-I}\ J\ K\ {-L}\ {-M}\ {-K}\ {-L}\ {-M}\ \circ)$.

Figure 3 and 4 gives the adjacency graph and the overlap graph of G. They show that G is an oriented valid-path genome whose adjacency graph contains $C = 3$ cycles. Therefore, the reversal distance is $d_{rev}(G) = n - C = 13 - 3 = 10$.

Algorithm 2, applied to the four oriented connected components of the overlap graph, provides the following reversal scenario of length 10 where reversed segments are underlined:

$(\circ\ A\ \underline{-B\ -A}\ -B\ C\ D\ -C\ -E\ -F\ -D\ G\ -H\ -G\ H\ I\ -J\ -F\ -E\ -I\ J\ K\ -L\ -M\ -K\ -L\ -M\ \circ)$

$(\circ\ A\ A\ B\ \underline{-B}\ C\ D\ -C\ -E\ -F\ -D\ G\ -H\ -G\ H\ I\ -J\ -F\ -E\ -I\ J\ K\ -L\ -M\ -K\ -L\ -M\ \circ)$

$(\circ\ A\ A\ B\ B\ C\ D\ -C\ -E\ -F\ -D\ G\ \underline{-H\ -G}\ H\ I\ -J\ -F\ -E\ -I\ J\ K\ -L\ -M\ -K\ -L\ -M\ \circ)$

$(\circ\ A\ A\ B\ B\ C\ D\ -C\ -E\ -F\ -D\ G\ G\ H\ H\ I\ \underline{-J\ -F\ -E\ -I}\ J\ K\ -L\ -M\ -K\ -L\ -M\ \circ)$

$(\circ\ A\ A\ B\ B\ C\ \underline{D\ -C}\ -E\ -F\ -D\ G\ G\ H\ H\ I\ I\ E\ F\ J\ J\ K\ -L\ -M\ -K\ -L\ -M\ \circ)$

$(\circ\ A\ A\ B\ B\ C\ C\ -D\ -E\ \underline{-F\ -D\ G\ G\ H\ H\ I\ I\ E}\ F\ J\ J\ K\ -L\ -M\ -K\ -L\ -M\ \circ)$

$(\circ\ A\ A\ B\ B\ C\ C\ \underline{-D\ -E\ -E\ -I\ -I\ -H\ -H\ -G\ -G}\ D\ F\ F\ J\ J\ K\ -L\ -M\ -K\ -L\ -M\ \circ)$

$(\circ\ A\ A\ B\ B\ C\ C\ G\ G\ H\ H\ I\ I\ E\ E\ D\ D\ F\ F\ J\ J\ K\ \underline{-L\ -M\ -K}\ -L\ -M\ \circ)$

$(\circ\ A\ A\ B\ B\ C\ C\ G\ G\ H\ H\ I\ I\ E\ E\ D\ D\ F\ F\ J\ J\ K\ K\ M\ \underline{L\ -L\ -M}\ \circ)$

$(\circ\ A\ A\ B\ B\ C\ C\ G\ G\ H\ H\ I\ I\ E\ E\ D\ D\ F\ F\ J\ J\ K\ K\ M\ M\ L\ \underline{-L}\ \circ)$

$(\circ\ A\ A\ B\ B\ C\ C\ G\ G\ H\ H\ I\ I\ E\ E\ D\ D\ F\ F\ J\ J\ K\ K\ M\ M\ L\ L\ \circ)$

The corresponding reversal scenario on the arrangement of the 14 synteny blocks is the following scenario, in which the reversal are denoted using the nomenclature of [4]:

(○ 14 −17 19 −15 18 −16 20 27 −21 −26 −28 22 −23 24 −29 −25 30 −33 35 −31 34 −32 36 ○)

↓ 2L(1)

(○ 14 15 −19 17 18 −16 20 27 −21 −26 −28 22 −23 24 −29 −25 30 −33 35 −31 34 −32 36 ○)

↓ 2L(2)

(○ 14 15 16 −18 −17 19 20 27 −21 −26 −28 22 −23 24 −29 −25 30 −33 35 −31 34 −32 36 ○)

↓ 2L(6)

(○ 14 15 16 −18 −17 19 20 27 −21 −26 −28 22 23 24 −29 −25 30 −33 35 −31 34 −32 36 ○)

↓ 2LR(7)

(○ 14 15 16 −18 −17 19 20 27 −21 −26 −28 22 23 24 25 29 30 −33 35 −31 34 −32 36 ○)

↓ 2LR(4)

(○ 14 15 16 −18 −17 19 20 21 −27 −26 −28 22 23 24 25 29 30 −33 35 −31 34 −32 36 ○)

↓ 2LR(8)

(○ 14 15 16 −18 −17 19 20 21 −27 −26 −25 −24 −23 −22 28 29 30 −33 35 −31 34 −32 36 ○)

↓ 2LR(5)

(○ 14 15 16 −18 −17 19 20 21 22 23 24 25 26 27 28 29 30 −33 35 −31 34 −32 36 ○)

↓ 2R(9)

(○ 14 15 16 −18 −17 19 20 21 22 23 24 25 26 27 28 29 30 31 −35 33 34 −32 36 ○)

↓ 2R(10)

(○ 14 15 16 −18 −17 19 20 21 22 23 24 25 26 27 28 29 30 31 32 −34 −33 35 36 ○)

↓ 2R(11)

(○ 14 15 16 −18 −17 19 20 21 22 23 24 25 26 27 28 29 30 31 32 −34 33 35 36 ○)

The reversals 2L(6), 2LR(8) 2LR(7) are 2-BD-reversal, and the seven other reversals of the scenario are 1-BD-reversal. In good agreement with biological results, the proposed scenario consists of 10 out of the 11 reversals proposed in the litterature to explain the evolution of the chromosome 2 of *D.mel* and *D.yak* [4]. Thus, the proposed ancestor of *D.yak* chromosome 2 is 10 reversals far from *D.yak*, and only 1 reversal far from *D.mel*. This results strongly supports the hypotheses of a slow evolution in the *melanogaster* species tree compared to the evolution in the *yakuba* species tree, raised by biological studies [15].
